# The histone variant H3.3 claims its place in the crowded scene of epigenetics

**DOI:** 10.18632/aging.101194

**Published:** 2017-03-10

**Authors:** Daniele Bano, Antonia Piazzesi, Paolo Salomoni, Pierluigi Nicotera

**Affiliations:** ^1^ German Center for Neurodegenerative Diseases (DZNE), 53127 Bonn, Germany

**Keywords:** aging, histone variant H3.3, histone chaperones, neuronal function, senescence

## Abstract

Histones are evolutionarily conserved DNA-binding proteins. As scaffolding molecules, they significantly regulate the DNA packaging into the nucleus of all eukaryotic cells. As docking units, they influence the recruitment of the transcriptional machinery, thus establishing unique gene expression patterns that ultimately promote different biological outcomes. While canonical histones H3.1 and H3.2 are synthetized and loaded during DNA replication, the histone variant H3.3 is expressed and deposited into the chromatin throughout the cell cycle. Recent findings indicate that H3.3 replaces the majority of canonical H3 in non-dividing cells, reaching almost saturation levels in a time-dependent manner. Consequently, H3.3 incorporation and turnover represent an additional layer in the regulation of the chromatin landscape during aging. In this respect, work from our group and others suggest that H3.3 plays an important function in age-related processes throughout evolution. Here, we summarize the current knowledge on H3.3 biology and discuss the implications of its aberrant dynamics in the establishment of cellular states that may lead to human pathology. Critically, we review the importance of H3.3 turnover as part of epigenetic events that influence senescence and age-related processes. We conclude with the emerging evidence that H3.3 is required for proper neuronal function and brain plasticity.

## INTRODUCTION

Eukaryotic cells compact their large genome into highly ordered chromatin structures within the nucleus. The dynamic nature of chromatin establishes the access to the genetic material and, as a consequence, influences a large number of biological processes, such as DNA replication, repair and transcription [1, 2]. The nucleosome is the basic packaging unit of chromatin. Each core nucleosome comprises 147bp of DNA bound to two copies of histones H2A, H2B, H3 and H4. Given the considerable stability of the nucleosome, eukaryotic cells employ a staggering array of interconnected molecular mechanisms that locally modify the electrostatic interaction between the highly basic histones and the negatively charged DNA molecules. In general, these epigenetic modifications work together and generate a code that ultimately determines the biological outcome [3, 4]. Among these regulatory processes, core and linker histones are subjected to a large pattern of posttranslational modifications (PTMs) that influence chromatin state and DNA accessibility [4-7]. Along with PTMs, the partial or complete disassembly of nucleosomes allows the exchange and degradation of pre-existing histone proteins, with the incorporation of newly synthesised histones onto chromatin that can eventually result in the resetting of previous epigenetic marks. In such a crowded molecular picture, recent exciting insights have uncovered the role of histone variants as key regulators of the chromatin structure. Compared to the canonical ones, histone variants contain limited amino acid differences or unique domains with distinct biochemical properties. Incorporation of histone variants confers variability to the chromatin and expands the repertoire of epigenetic marks in a functional alphabet that controls genome plasticity and dynamics [4]. Throughout evolution, eukaryotes adopted a network of highly conserved proteins that buffer the positive charges of histones, maintaining their solubility and, therefore, avoiding aberrant interactions with other cellular components. These dedicated proteins are generally known as histone chaperones and effectively control histone supply and chromatin dynamics [8]. Here, we focus essentially on the histone variant H3.3 and the associated complexes that selectively regulate its homeostasis and dynamics. In addition, we review the importance of histone H3.3 turnover in human health as well as its emerging role in disorders.

### The relevance of histone variant H3.3 in physiology and pathology

**Figure 1:**
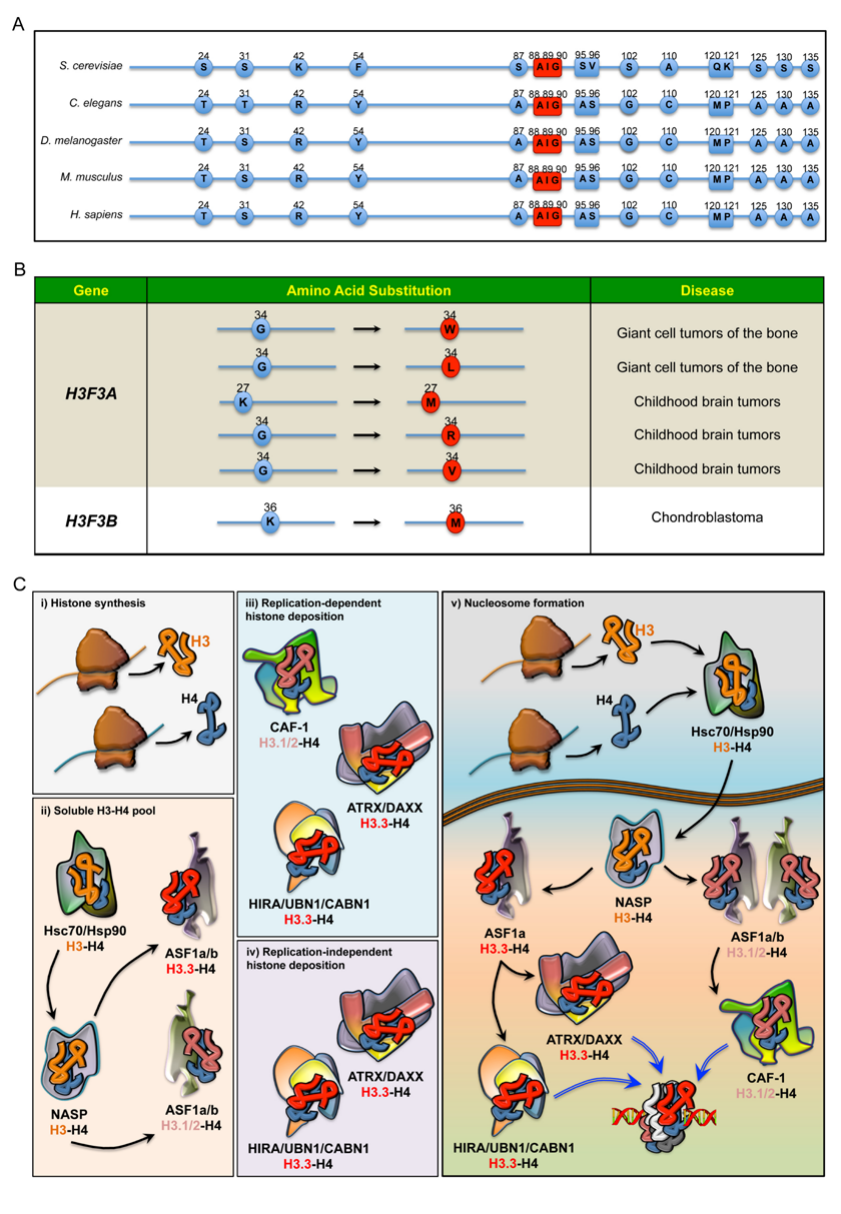
(**A**) Sequence alignment comparing the differences in the amino acids of H3.3 in five species. In red: the evolutionarily conserved amino acids that, along with Ala87, coordinate the binding to dedicated histone chaperones. (**B**) Annotated somatic mutations in *H3F3A* and *H3F3B* causally linked to tumours in humans. (**C**) Regulation of histone H3 pool in the cell. i) Newly synthesised histone proteins H3 and H4 ii) associate with Hsc70 and Hsp90, which determine the stability and degradation rate of H3-H4 dimers. In the nucleus, the binding to NASP controls the supply of soluble H3-H4 to ASF1a and ASF1b. H3-H4 dimers are then handed over to ASF1a/b, iii) which then transfers H3.1/2-H4 dimers to CAF-1, iv) whereas ASF1a transfers H3.3-H4 dimers to both HIRA/UBN1/CABN1 and ATRX/DAXX for loading onto the chromatin. v) Schematic overview of histone chaperones binding H3.1/2 and H3.3, from their synthesis to their deposition onto the chromatin.

The H3 family comprises seven identified human H3 variants: the two canonical H3.1 and H3.2 proteins, the replication-independent H3.3, the centromere protein A (CENP-A), the testis-specific histone H3t and the primate-specific H3.X and H3.Y. As in other metazoans, human genes encoding canonical H3.1 and H3.2 are organized in multi-copy clusters with no introns. Apart from a few recently described exceptions [9], mature mRNA transcripts do not contain polyadenylated tails and terminate with a highly conserved stem-loop that enhances transcript instability and degradation. The expression levels of these histones peak during DNA replication when they are incorporated into chromatin in a DNA-synthesis coupled manner [10, 11]. This distinct expression pattern of intronless transcripts allows the biosynthesis of histone H3 in large amounts for the proper assembly of nucleosomes during S-phase [10, 11]. Conversely, the intron-containing *H3F3A* and *H3F3B* genes, which are transcribed into post-transcriptionally polyadenylated mRNAs, encode identical H3.3 proteins in a replication-independent fashion in embryonic as well as in differentiated cells [12-14]. The vast majority of higher eukaryotes express canonical as well as replication-independent histone H3 variants, whereas *Saccharomyces cerevisiae* has only one archaic H3.3-like protein that is deposited in both manners. In multicellular organisms, the H3.3 protein sequence differs from the canonical ones in no more than five amino acids. Compared to H3.2, Ala31 is substituted with a phosphorylatable residue of Ser or Thr in H3.3, while the globular core of H3.3 comprises the amino acids Ala87, Ile89 and Gly90 that confer the unique biochemical affinities to the certain motif of binding proteins (Figure 1A). Along with other residues, Ser31 is highly phosphorylated during mitosis, although its distribution pattern is observed primarily in chromosomal regions flanking the centrosome [15]. Moreover, the presence of a hydroxyl side-chain at position 31 seems important for signaling processes, as it generates repulsive electrostatic forces that interfere with the activity of enzymes selectively recognizing or modifying the important Lys27 residue at the amino-terminal tail [16]. To a similar extent, the substitution of the three amino acids in the globular core of H3.3 disrupts the specialized recruitment of distinct histone-binding factors. Indeed, Gly90 determines hydrogen bonds and hydrophobic interactions that uniquely anchor H3.3 to the binding pockets of dedicated histone chaperones [17, 18]. Conversely, substituting any of these three amino acids in canonical H3 with their H3.3 counterparts is enough to cause the protein to be loaded in a replication-independent manner in *Drosophila melanogaster* [19], further highlighting their importance in recruiting specific histone chaperones.

Since *S. cerevisiae* has only one histone H3.3-like variant (Figure 1A), one plausible hypothesis is that metazoans evolved new H3 isoforms from duplication of this archaic H3.3-like gene. In budding yeast, the replication independent incorporation of H3 onto transcriptionally active genes promotes gene transcription. This functional association with actively transcribed chromatin has been maintained in multicellular organisms, and complete loss of H3.3 results in phenotypes of varying severity across the eukaryotic kingdom. Two genes encode for H3.3 protein in both post-mitotic and proliferating germ cells of *Caenorhabditis elegans* [20]. Overall, H3.3 deficient animals are viable and fertile, though they exhibit an increased susceptibility to stress [20, 21]. In *Drosophila melanogaster*, on the other hand, complete loss of H3.3 results in reduced viability and complete sterility in both males and females, though both copies of H3.3 must be deleted to provoke this phenotype [22, 23]. Not surprisingly, a more complex array of phenotypes has been observed in vertebrates. In *Xenopus laevis*, knockdown of H3.3 results in late gastrulation defects [24], while *h3f3a* mutant zebrafish have an almost complete lack of head skeletal structures [25]. In rodents, a hypomorphic mutation as well as the knockout of *H3f3a* results in incomplete embryonic lethality, with the surviving animals exhibiting reduced growth rate and partial male sterility [26, 27]. The phenotype of *H3f3b* homozygous knockout mice seems to be even more severe. Contrary to the redundancy observed in invertebrates, inactivation of both *H3f3b* alleles causes foetal death before or immediately after delivery [27], although there are some discrepancies with a previously published model in which a few surviving *H3f3b* mutants could reach adulthood [28]. Notably, ablation of both *H3f3a* and *H3f3b* leads to premature oocyte death [27], further highlighting the importance of H3.3 in development.

An emerging body of literature has indicated the contribution of H3.3 to human diseases. New exciting findings have identified recurrent dominant H3.3 mutations in childhood brain tumours (Figure 1B). Two sets of heterozygous mutations in the *H3F3A* gene (and to a lesser extent canonical H3 genes) cause N-terminal tail amino acid substitutions of Lys27 (K to M) and Gly34 (G to R or V), which have been proposed to drive brainstem and forebrain high-grade glioma (HGG), respectively [29-31]. Moreover, missense *H3F3B* mutations leading to distinct Gly34 substitutions have been identified in most cases of giant cell tumour of the bone, whilst the K36M mutation defines chondroblastoma [29, 30]. Although the molecular mechanisms underlying these aggressive tumours at a young age remain elusive, one possible explanation is the establishment of a permissive chromatin landscape. In this respect, both K27M and K36M mutations work through gain-of-function mechanisms, leading to inhibition of the respective methyltransferases and global reduction in H3K27me3 and H3K36me3 levels, respectively [32]. Consistent with these findings, expression of H3.3K27M in human ES cell-derived neural precursor cells (hNPCs) affects the transcriptional regulation of many genes and induces oncogenic transformation in cooperation with p53 loss and a constitutively active PDGFRA mutant [33]. However, this model is based on an *in vitro* transformation after prolonged culture of hNPCs and fails to induce HGG upon orthotopic transplantation [33]. Much less is known about Gly34 mutations, but it has been proposed that they may alter the methylation pattern at Lys36 (H3K36me3), driving the aberrant expression of genes that ultimately promote neoplasms [32, 34, 35]. Further studies are necessary to confirm this model.

### HIRA and ATRX/DAXX complexes: two specialized histone chaperones for H3.3 turnover

Nucleosome assembly requires a sequential incorporation of heterodimers onto DNA strands, with H3-H4 dimers that promote the formation of the intermediate core particle [36]. The deposition of histone variants is a crucial aspect of chromatin dynamics and is dependent on an array of dedicated molecular machineries (Figure 1C). To prevent misfolding and degradation, newly synthesized cytosolic H3 histones associate with heat-shock proteins, which are then transferred to nuclear autoantigenic sperm protein (NASP). Homologue to the yeast Hif1p, NASP coordinates the assembling of stable H3-H4 dimers and contributes to the maintenance of a stable pool of chromatin components ready to be supplied in response to the cell's need [37, 38]. Once part of the complex, H3-H4 dimers can be handed to the antisilencing function 1 a and b proteins (ASF1a and ASF1b) [8, 39-42], two evolutionarily conserved orthologues that coordinate H3.1/2 and H3.3 deposition through preferential nucleosome assembly pathways. Although functionally related and partially redundant for H3-H4 binding, ASF1a and ASF1b are found to associate with histone regulator A (HIRA) and chromatin assembly factor-1 (CAF-1) with different degrees of affinity [36, 43]. While CAF-1 cooperates with both isoforms for H3.1-H4 deposition, HIRA preferentially binds ASF1a and competes with CAF-1 in a mutually exclusive fashion and using analogous specialized motifs [41, 43]. Since ASF1 does not discriminate between H3.1/2-H4 and H3.3-H4 dimers, it seems to function as a non-specific histone carrier that prevents tetramerization of H3-H4 and recycles evicted histones, providing dimers to CAF-1 or HIRA subunits that selectively determine the H3 variants according to their unique amino acids [18, 36, 38, 44, 45]. Consistently, in vivo evidence suggests that ASF1 depends on CAF-1 and HIRA for histone deposition, despite its ability to transfer purified histones to naked DNA [46, 47]. Thus, the use of H3.3 rather than canonical H3 critically depends on the binding affinity of certain adaptor proteins. In higher organisms, two multisubunit histone chaperones guide H3.3 deposition onto chromatin: HIRA/UBN1/CABIN1 (herein as HIRA) and ATRX/DAXX.

#### The HIRA/UBN1/CABIN1 complex

The HIRA complex comprises HIRA, the associated protein Ubinuclein-1 (UBN1) and calcineurin-binding protein 1 (CABIN1) [18, 48, 49], with ASF1a acting as a transient donor of H3.3-H4 dimers [36, 43]. It is an evolutionarily conserved assembly that regulates deposition and eviction of H3.3-H4 dimers in a DNA-replication independent manner [36, 50]. The *S. cerevisiae* Hir1p and Hir2p share homologous domains with HIRA [51-53], while Hir3 and Hpc2 are orthologues of CABIN1 and UBIN1, respectively [48, 54]. As a non-redundant component of the complex, UBN1 determines the specificity toward H3.3-H4 dimers, with H3.3 Gly90 that coordinates the binding along with the other amino acid residues within a domain highly conserved from yeast to mammals (Figure 1A) [18]. The *S. cerevisiae* HIR complex participates in transcriptional regulation, elongation rate and establishment of silenced chromatin domains in a replication-independent manner. In *Drosophila melanogaster*, HIRA is required for the deposition of H3.3 in the decondensed sperm pronucleus, and flies lacking HIRA only possess maternal chromosomes and are thus embryonic lethal [55]. However, HIRA is not essential for the deposition of H3.3 at other stages of *Drosophila* development, indicating that it is not the only H3.3-specific chaperone present in flies. In *Xenopus laevis*, H3.3 incorporation during development is also HIRA-dependent, and knockdown of HIRA in embryos phenocopies the knockdown of H3.3 [24]. In mammals, HIRA-mediated H3.3 deposition is critical for early embryonic development and cell pluripotency. In proliferating cells, the HIRA complex occupies thousands of loci across the genome and regulates the functional properties of actively transcribed genes [56], since it controls the presence of the RNA polymerase RNA pol II at transcription sites and regulatory elements [57]. According to very recent evidence, HIRA association to chromatin, as well as HIRA-mediated nucleosome assembly of H3.3, is dependent on replication protein A (RPA), a single-stranded DNA binding protein previously described as a master regulator of DNA replication and repair [58, 59]. Consistent with a role in H3.3 deposition onto regulatory elements and promoters, downregulation of RPA affects the recruitment of HIRA and alters gene transcription. Mechanistically, these new findings describe RPA as a new critical factor that, in an evolutionarily conserved manner, regulates nucleosome assembly through the binding of different H3 histone chaperones [58, 59].

In the pathological context, *HIRA* was initially associated with DiGeorge syndrome, as the *HIRA* gene lies within the q11 region of chromosome 22, which is deleted in these patients [52]. However, no further evidence supports the direct role of HIRA in this syndrome, since many other genes are also located within this deletion. To our knowledge, no distinct human pathologies have been specifically linked to annotated mutations of HIRA complex subunits. However, it would not be surprising if they were found in some tumours, given HIRA role in H3.3 deposition, and the association between H3.3 mutations and some forms of cancer.

#### ATRX/DAXX complex

The α-thalassemia/mental retardation syndrome X-linked protein (ATRX) is an ATP-dependent chromatin remodelling factor and belongs to the family of SNF2-related ATPases [60]. Like other helicase subunits of eukaryotic SWI/SNF multiprotein complexes, ATRX modifies nucleosome composition upon recruitment to distinct targeted sites. Its localization at telomeres and pericentric heterochromatin initially suggested a potential role in the maintenance of silent chromatin [61-63]. Along with ATRX, the death-domain associated protein DAXX takes part in H3.3 deposition onto chromatin [44, 64, 65]. Similar to the HIRA complex, DAXX anchors H3.3 using a solvent-filled pocket that coordinates the AAIG motif, with an affinity for H3.3-H4 dimers that allows ASF1 displacement [17]. The association of DAXX with ATRX results in the ATP-dependent remodelling of chromatin and H3.3 deposition at defined genomic regions in a replication-independent fashion [44, 66, 67]. Recent evidence indicates that ATRX recognizes H3K9me3 and unmodified H3K4 via its chromodomains and binding to the heterochromatin protein HP1 [68]. This promotes the eviction of the histone variant macroH2A1 at genes and intergenes, as demonstrated in human-derived cells in which ATRX deficiency causes an accumulation of macroH2A1 at subtelomeric regions [69]. In association with DAXX, ATRX controls the deposition of histone variant H3.3 at pericentric heterochromatin, telomeres and transcriptional start sites in both dividing and differentiated cells [44, 64, 70-72]. Although H3.3 has usually been associated with active promoters and regulatory regions of expressed genes [73-76], it seems that ATRX/DAXX critically controls H3.3 deposition on silenced methylated alleles, maintaining epigenetic modifications such as H3K9me3 [72]. This effect may prevent the loss of epigenetic memory during transcription and inducing aberrant gene expression of heterochromatin loci. Similarly, ATRX/DAXX is critical for the deposition of H3.3 onto endogenous transposable elements, such as endogenous retroviral elements (ERVs) in mouse embryonic stem cells [77]. In this case, H3.3-dependent nucleosome turnover sustains H3K9me3 and maintains silenced ERVs through the recruitment of the co-repressor KRAB-associated protein-1 (KAP1). Conversely, CAF-1-mediated replacement of H3.3 with canonical H3.1 and H3.2 keeps retrotransposons in a silenced state during preimplantation of mouse embryos [78]. Since transposable element activity alters the expression of a large number of genes and may have contributed to the evolution of primates, these findings support the role of H3.3 turnover in the maintenance of genomic stability and somatic mosaicism within tissues. In the context of differentiated neurons, while the HIRA complex primarily mediates the activity-dependent deposition of H3.3 onto chromatin [74, 79], ATRX/DAXX may contribute to a smaller set of genes in the nervous system. At least in primary dissociated cortical neurons, ATRX/DAXX promotes the incorporation of H3.3 at promoters and enhancers of immediate early genes upon membrane depolarization [70].

From the clinical standpoint, ATRX was originally identified as the gene responsible for α-thalassemia X-linked mental retardation syndrome, a rare and inherited intellectual disability which is also characterized by developmental delays, distinctive craniofacial features and skeletal abnormalities, genital abnormalities and anaemia [67, 80, 81]. Various different mutations in the ATRX gene have been recently linked to this syndrome [81]. However, considering the crucial role of chromatin dynamics in cell proliferation and differentiation, it is not surprising that ATRX and DAXX have also been unequivocally associated with certain human tumours. In this respect, inactivating mutations in either of the two encoding genes are frequently observed in neuroendocrine tumours of the pancreas [82-84]. Moreover, ATRX is mutated in astrocytic tumours [85], HGG and in neuroblastoma, whilst DAXX deficiency has been linked to a few cases of paediatric HGG, with a mutually exclusive pattern to ATRX mutations [30, 65, 86]. Notably, ATRX and DAXX mutations are almost exclusively associated with H3.3 mutations in paediatric HGG, and ATRX is also mutated in wild type H3.3 adult astrocytoma [30]. Together, these studies underscore the importance of ATRX/DAXX in chromatin remodelling and in the pathogenesis of clinically relevant human diseases.

### H3.3 in senescence and aging

Cellular senescence is the irreversible arrest of eukaryotic cell proliferation and the development of an altered senescence-associated secretory phenotype, occurring both *in vitro* and *in vivo* [87]. From an evolutionary viewpoint, senescence limits the proliferative capacity of cells and therefore acts as a tumour suppressor. It is an energetically demanding process, since senescent cells have a significant metabolic shift toward “aerobic glycolysis” and reduced mitochondrial oxidative phosphorylation [88]. More importantly, the survival of senescent cells can be deleterious for normal tissue homeostasis, since non-proliferating cells limit the regenerative capacity of the body and affect the function of neighbouring cells through their secreted proinflammatory factors. Such a causal role in the loss of organismal fitness has been recently demonstrated in mice. Indeed, genetic manipulation that reduces the number of senescent-positive cells delays many age-related phenotypes and has a positive effect on mouse survival [89]. This and other evidence supports the idea that senescence contributes to several aspects of aging and age-related disorders.

Mechanistically, senescence is driven by a complex cellular response. In cultured cells as well as in aged tissue, morphological alterations include the formation of large domains of compacted chromatin generally known as senescence-associated heterochromatin foci (SAHF) [90, 91]. These chromatin modifications are associated with widespread changes in gene expression that seem to contribute to cell cycle arrest and the consequent senescence program [91]. Despite the presence of repressive marks, such as the transcription-silencing histone variant macroH2A, and the HIRA/ASF1-dependent formation of SAHF [90], senescent cells appear to maintain a very dynamic chromatin landscape in which deposition of newly synthesized histones occurs constantly. When compared to proliferating cells, senescent cells express a subset of replication-dependent histones that are necessary for physiological nucleosome turnover. The incorporation of these canonical histones is coupled to HIRA-dependent deposition of the histone variant H3.3 [92]. In models of oncogene-induced and replicative senescence models, it seems that deposited H3.3 can be cleaved at the N-terminal in the nucleus by the lysosomal protease Cathepsin L1 [93]. The proteolytic processing of H3.3 removes the histone posttranslational modifications (PTMs) that critically control the expression of cell cycle regulators. Ectopic expression and HIRA-mediated loading of cleaved H3.3 is sufficient to promote cellular senescence. Notably, senescent cells lacking HIRA exhibit a marked decreased of H4K16ac at many promoters of transcribed genes across the genome, further confirming the prominent role of HIRA in regulating highly compacted, transcriptionally silenced chromatin [92]. The maintenance of H4K16ac in senescent cells prevents promoter silencing and functions as a tumour suppressor. Consistently, HIRA deficiency profoundly affects chromatin structure and sensitizes senescent cells to oncogene-induced neoplasia *in vivo* [92]. Overall, the contribution of histones and histone chaperones to cellular senescence remains an exciting area of on-going research. In dividing cells, it is known that senescence programs and aging are associated with decreased biosynthesis of histones and global changes in chromatin structure. Consistent with the role of chromatin maintenance in age-related pathways, diminished histone supply induces replicative senescence in human fibroblasts and reduces replicative life span in yeast [94, 95]. Conversely, overexpression of histone H3-H4 sustains a dynamic nucleosome turnover that prevents aberrant transcription and genomic instability, delaying age-related processes in dividing cells. Based on these findings, it would be very much of interest to define which chromatin structures are causally linked to aging in multicellular organisms. In the long run, the characterization of the underlying molecular mechanisms might provide valuable therapeutic targets in age-related human disease.

Aging is a multifactorial process that progressively affects the physiological integrity of various tissues and ultimately leads to the fitness loss of an organism [96]. Although an inevitable part of life, some age-related traits can, at least in principle, be delayed, as demonstrated in various model organisms. Many pharmacological and genetic interventions increase healthy longevity in an evolutionarily conserved manner across a wide range of species [97-99]. Based on our current understanding, most of the pro-survival signaling pathways converge on a common signature that includes a general metabolic rewiring and a prominent transcriptional regulation of stress-response genes. In this regard, one of the first described and perhaps the most prominent example is the lifespan-extending effect of the insulin/IGF-1 signaling pathway [100]. Diminished activity of the insulin/IGF-1/DAF-2 receptor as well as the downstream target phosphatidylinositol 3-kinase PI3K/AGE-1 extends the lifespan of the nematode *Caenorhabditis elegans* [101, 102]. The subsequent nuclear translocation of FOXO/DAF-16 induces the recruitment of the ATP-dependent chromatin remodelling SWI/SNF complex onto a large number of promoters, thus maintaining a permissive chromatin landscape that supports a broad transcriptional response [103, 104]. Consistent with these findings, we have recently demonstrated that H3.3 loss-of-function significantly compromises the DAF-16-mediated lifespan-extending programs [21]. In *daf-2* mutant nematodes, lack of H3.3 perturbs the expression of a large number of genes, resulting in a much shorter lifespan. Notably, the lifespan reduction is not limited to insulin/IGF-1/DAF-2 mutant animals, since we have showed that H3.3 deficiency alters the longevity of germline-deficient as well as mitochondrial mutant animals. Consistent with the evidence in a *daf-2* mutant background, H3.3 loss-of-function compromises the expression of those genes that critically support stress resistance and metabolic rewiring induced by mild mitochondrial impairment. Thus, since H3.3 dictates the survival rate of other long-lived mutants, we propose H3.3 homeostasis as a common key regulator of a permissive chromatin landscape that enables the proper engagement of transcriptional programs that ultimately promotes longevity. Moreover, as H3.3 is the critical H3 variant in postmitotic cells [21, 74], it is plausible that canonical histones cannot compensate for the lack of this unique protein, with a negative functional impact on genomic stability. As a consequence, it is likely that the molecular machinery regulating H3.3 turnover and dynamics may be critical epigenetic mediators that control the chromatin state underlying age-related processes in metazoans.

### H3.3 and its contribution to neuronal function

The central nervous system of a multicellular organism is able to store long-lasting memories that can influence sophisticated behaviours critical for animal survival. Moreover, it orchestrates decision-making and adaptation responses to a wide range of environmental stimuli. This plasticity depends on signaling cascades that control neuronal structures at the morphological and functional level [105]. Ample evidence indicates that activity-dependent changes in gene expression underlie neuronal plasticity [106]. In this complicated scenario, epigenetic modifications of the chromatin state establish transcriptional profiles in a cell-type specific manner. Arrays of nucleosome-modifying complexes as well as synergistic PTMs of histones and DNA determine chromatin compaction and the switching between silent and transcriptionally active chromatin [75, 106]. While histone PTMs have been a matter of considerable interest for decades [5, 6, 106], only recent studies have emphasized the mechanistic importance of histone variants and nucleosome turnover in the context of neuronal plasticity, behaviour, cognition and memory consolidation [107]. Starting from an accepted view of nucleosome cores being relatively stable in differentiated neurons, a series of remarkable experiments demonstrate the essential role of H3.3 in the brain of adult animals [74]. Unlike in embryonic cells, H3.3 starts to accumulate after birth in the chromatin of glia and post-replicative neurons, reaching saturation levels within a relatively short period of time. In adulthood, H3.3 replaces almost the entire pool of canonical H3.1 and H3.2 in the neuronal genome. Strikingly, there is a dynamic turnover of H3.3-containing nucleosomes throughout the lifetime of mice as well as humans, suggesting a rate of histone exchange previously underestimated in the central nervous system. This constant activity-induced remodelling occurs in a proteasome-dependent manner and seems to be dissociated from posttranslational modifications that actively mark transcribed chromatin domains. At the cellular level, the reduction of functional excitatory and inhibitory synapses due to H3.3 downregulation correlates with aberrant plasticity-associated gene expression profiles [74]. Consistent with these findings, animals with decreased expression of H3.3 in the hippocampus exhibit impaired long-term memory, underlying the importance of H3.3 turnover in cognitive functions [74]. Despite the limited current knowledge, recent studies have suggested an involvement of H3.3 in psychiatric disorders. One prominent example is found in individuals with major depressive disorders (MDD), who show increased H3.3 expression in the nucleus accumbes (NAc), one of the key components of the reward system [108]. Consistent with an implication in MDD, depression-related upregulation of H3.3 is modulated in patients following antidepressant treatment [79]. The mechanistic link between H3.3 dynamics and psychiatric disease is also demonstrated in mouse models of chronic social stress, since specific H3.3 knockdown in the NAc of stressed mice is sufficient to inhibit depressive-like behaviours and the associated transcriptional patterns [79]. Together, this evidence opens new attractive venues of research in the field of psychiatric illness.

### PERSPECTIVES AND CONCLUSIONS

Following the advent of whole exome sequencing methods, many disease-causing mutations affecting chromatin dynamics have been linked to neurological syndromes characterized by intellectual disability. For example, *de-novo* dominant mutations of the ATP-dependent chromatin remodelling SWI/SNF complex have been found in various forms of developmental disorders, including autism [109]. It thus follows to ask, does H3.3 also play a role in neurological disorders? Since ATRX is disrupted in X-linked alpha-thalassemia mental retardation syndrome, we can infer that H3.3 is likely crucial for the establishment of an appropriate chromatin landscape that allows brain plasticity. However, since H3.3 chaperones could also have H3.3-independent functions, it is fundamental to determine whether H3.3 itself plays any role in age-related brain disorders. In this respect, it is currently unknown whether H3.3 biology is altered in common neurodegenerative disorders, including certain forms of dementia. It is tempting to speculate that the global dysregulation of neuronal activity and proteostasis during aging may impair H3.3 biology in the central nervous system. In turn, this would have a significant impact on gene expression programs, with consequences in the cognitive capacity of an individual. To further elucidate H3.3's specific involvement in neurological disorders, animal models need to be generated which circumvent H3.3's crucial role in development. The current body of knowledge available also begs the question: is H3.3 the only histone variant important in neuronal function and neurodegeneration? As H3.3 is not the only replication-independent histone variant, it stands to reason that other histone variants with similar properties might play a parallel and/or an equally important role. Prior findings have indicated that other histone variants are particularly important for proper neuronal function. In this regard, dynamic regulation of H2A.Z exchange is associated with activity-induced gene expression in established experimental paradigms of memory consolidation [110]. Based on this evidence, it seems that various histone variants, their deposition and the consequent nucleosome turnover represent an additional layer of complexity in the epigenetic regulation of specific patterns of genes that ultimately establish neuronal function and brain plasticity.

In conclusion, given the contribution of H3.3 to age-related signaling processes [21], we expect that future studies will elucidate whether and how H3.3 turnover, along with other histone variants, contribute to the onset of sporadic forms of brain pathologies, including Alzheimer's and Parkinson's disease. Undoubtedly, this new frontier of epigenetics will likely clarify the role of histone variants in the aging process and associated diseases, thus providing new insights into the pathogenesis of many debilitating human disorders.
